# Evaluation of the effect of probiotic as add-on therapy with conventional therapy and alone in malaria induced mice

**DOI:** 10.1186/s13104-021-05661-1

**Published:** 2021-06-30

**Authors:** Eshani Mahajan, Shweta Sinha, Alka Bhatia, Rakesh Sehgal, Bikash Medhi

**Affiliations:** 1 Department of Pharmacology, Postgraduate Institute of Medical Education & Research, Research Block B, 4th Floor, Room no 4043, Chandigarh, 160012 India; 2 Department of Medical Parasitology, Postgraduate Institute of Medical Education and Research, Chandigarh, 160012 India; 3 Department of Experimental Medicine and Biotechnology, Postgraduate Institute of Medical Education and Research, Chandigarh, 160012 India

**Keywords:** Chloroquine, Probiotic, Malaria, *Lactobacillus casei*

## Abstract

**Objective:**

Chloroquine is used as a conventional drug therapy for the treatment of malaria. The existence of resistance to chloroquine shown among various species of *Plasmodium* leads to the search for more efficacious therapy to treat malaria. Probiotic (*Lactobacillus casei*) has been tried as an add-on therapy with chloroquine. Probiotics are ingested microorganisms associated with a beneficial effect on humans and other species. The study was done to check the efficacy of *L. casei* as an add-on therapy along with conventional drug therapy (chloroquine) to treat malaria.

**Results:**

Probiotic in combination with chloroquine showed complete suppression in parasitemia rate. Representation of parasitemia rate was done using mean ± SD. p < 0.05 is considered as statistically significant. The results showed a reduction in parasitemia with probiotic treatment, which was further confirmed through histological observation of two major organs, the liver and spleen. Interestingly, further suppression of parasitemia and hemosiderosis was observed when probiotic was given along with chloroquine.

**Supplementary Information:**

The online version contains supplementary material available at 10.1186/s13104-021-05661-1.

## Introduction

Malaria is among one of the deadliest threats for the human species which is caused by various strains of malaria parasites e.g., *Plasmodium falciparum, Plasmodium vivax, Plasmodium ovale, Plasmodium malariae, and Plasmodium knowlesi*. The risk of developing malaria is about 3.3 billion all over the world [1]*.* The immune response plays a major role in the pathophysiology of malaria. Various different immune proteins, which are released during the parasite attack can prohibit the growth of the same. Moreover, some signaling pathways act against the parasite e.g., Tolls like receptor, signal transducers and activator of transcription pathway (STAT), and Janus kinase pathway (JNK) [2]. Chloroquine was the choice of drug for the treatment of *P. falciparum* malaria, but these days there is an emergence of resistance among *P. falciparum* species [3]. Microorganisms that are believed to provide health benefits to the consumer are known as probiotics. These are generally gram-positive bacteria which are mainly isolated from gut microflora and are known to provide an enhancement in the immune response to the consumer. Probiotics provide strain-specific effectiveness as it has immune stimulatory properties against various pathogens and has ability to modulate intestinal microorganisms. Probiotics have shown their effects on various epithelial cells, Payer's patches cells, and immune cells [4]. The result of this interaction is an increase in the number of antibodies such as, IgA and IgM [5]. Past studies had shown that gut microbiota correlates with the severity of malaria parasite infection. Moreover, probiotics have beneficial effects against malaria parasite infection [6]. Several studies which have specific protocol towards understanding the molecular mechanism of probiotics needed to be done which involves its clinical application as well. The present study evaluates the effects of probiotic (*Lactobacillus casei*) on parasitemia count, histopathological changes in malaria-infected mice and tried to demonstrate the synergistic effect of this probiotic, along with chloroquine (conventional drug therapy) which can further lead to the development of fixed-dose probiotic combinations for the treatment of malaria.

## Main text

### Materials and methods

#### Study area

The study was designed as a single experimental, observational study which was conducted in the Department of Parasitology and Department of Pharmacology at the PGIMER, Chandigarh, India, for a duration of 1 year after taking approval from the Institutional Animal Ethics Committee (IAEC), PGIMER, Chandigarh, vide Ref No. 81/IAEC/521. Balb/c mice (n = 32) were obtained from Institutional Central Small Animal Facility, PGIMER, Chandigarh, and were allocated to the following equally sized (n = 8) groups as: (i) Group I (non-infected) (ii) Group II (Infected group) (iii) Group III (*P. berghei* + *L. casei*) (iv) Group IV (*P. berghei* + *L. casei* + chloroquine)**.** Laboratory standard cages were used for housing of the mice and acclimatized for 7 days prior to the start of experiments. Standard livestock feed and clean drinking water were given to them. This study was conducted according to the Committee for the Purpose of Control and Supervision of Experiments on Animals (CPCSEA) guidelines.

#### Treatment procedure

PBS was administered to the mice of Group I (non-infected) for four consecutive days, while *P. berghei* strain NK-65 infection was given to all other groups except Group I. The mice were infected via injecting 0.2 mL suspension of 10 parasitized erythrocyte intraperitoneally as described previously [7]. Samples of blood were taken from the tail of mice. Parasitemia ra^6^te were determined after preparation of thin blood smear following Giemsa staining. *P. berghei* infection was given to the second group. *L. casei* along with *P. berghei* was given to the third group. Chloroquine was administered to the mice at the dose of 15 mg/kg once a day for four consecutive days in the fourth group [7]. After completion of all the experimental procedures, animals were sacrificed by giving anaesthesia followed by cervical dislocation. This euthanasia procedure was done according to CPCSEA guidelines.

#### Determination of parasitemia

For the determination of the baseline parasitemia, thin smear was made using the blood sample [8]. By using a light microscope at 1000× magnification, we have checked the parasitemia rate by counting infected erythrocytes (parasitized) out of the 200 erythrocytes (both infected and non-infected) per field and counting continues till 10 fields. The following formula used for the calculation of parasitemia rate/percentage:

$$PR\left( {Parasitemia\,Rate} \right)\;\; = \frac{{Total\,number\,of\,pRBC\left( {parasitized\,RBC} \right)}}{{Total\,number\,of\,RBC\left( {infected\,and\,non-infected} \right)}} \times 100$$

#### Histopathology

For histopathological studies, the animals were sacrificed by cervical dislocation on the fourth day of respective treatment and the organs were harvested. Pathological changes were observed in two organs, i.e., the liver and spleen. Tissue from each group was fixed in 10% formalin and embedded with paraffin. After routine processing, paraffin sections from each tissue were cut into 5 µm thickness and stained with hematoxylin and eosin. The photomicrographs of the relevant stained sections were taken with the aid of a light microscope [9]. The following scores were used to grade the degree of histopathological changes or lesions observed in the organs: not observed ( −), mild ( +), moderate (+ +), and severe (+ + +).

#### Data analysis

Data analysis was done using statistical software, i.e., SPPS Version 21. Parasitemia count is represented as mean ± SD. Post hoc analysis was done by using ANOVA and Bonferroni multiple comparison tests for the comparison of means. p < 0.05 is considered statistically significant.

### Results

**Fig. 1:**
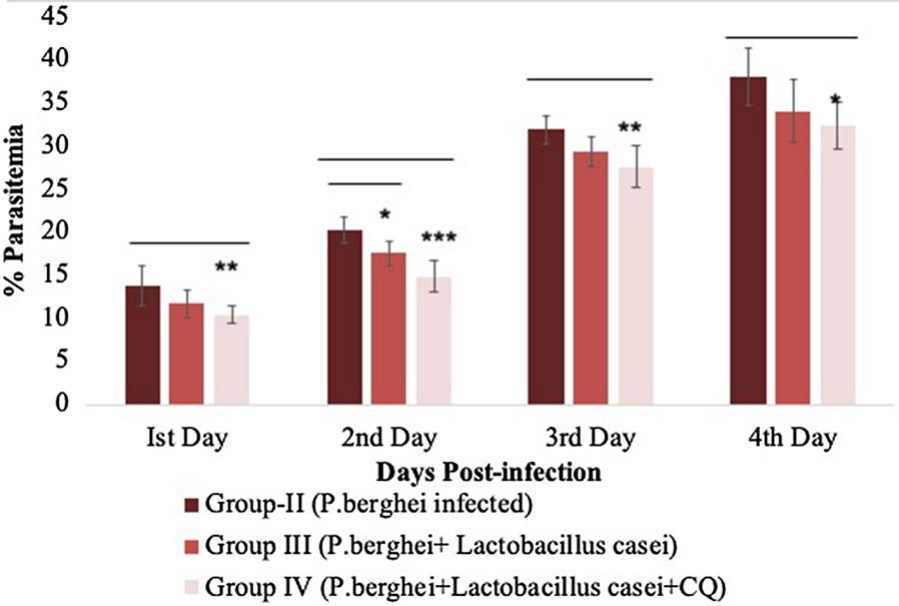
Graph showing percentage Parasitemia. The data are represented as mean ± SD. Statistical significance of data are given as *p < 0.05; **p < 0.01;*** p < 0.001

Parasitized RBCs were seen using light microscopy on the fourth day of inoculation by using the Giemsa staining technique on the microscopic slides. On the first day as compared to Group II (*P. berghei* treated*)*, Group IV (*P. berghei* + *L. casei* + chloroquine) has shown a statistically significant decrease (p < 0.01) in parasitemia %, On second day Group III (*P.berghei* + *L. casei*) and Group IV (*P. berghei* + *L. casei* + chloroquine) has also shown a statistically significant decrease in parasitemia % (p < 0.05 and p < 0.001) as compared to group II (*P. berghei*) while on day third, Group IV has shown statistical significant (p < 0.01) decrease in parasitemia % as compare to group II. Finally, on the last fourth day group IV has shown a statistically significant decrease in parasitemia % (p < 0.05) as compared to the *P. berghei* treated group, shown in Fig. 1. Overall, it has been shown that there is a reduction in parasitemia % when *L. casei* alone and *L. casei* along with chloroquine was given as compared to the infected group (*P. berghei* treated), shown in (Additional file 1: Figure S1).

**Fig. 2:**
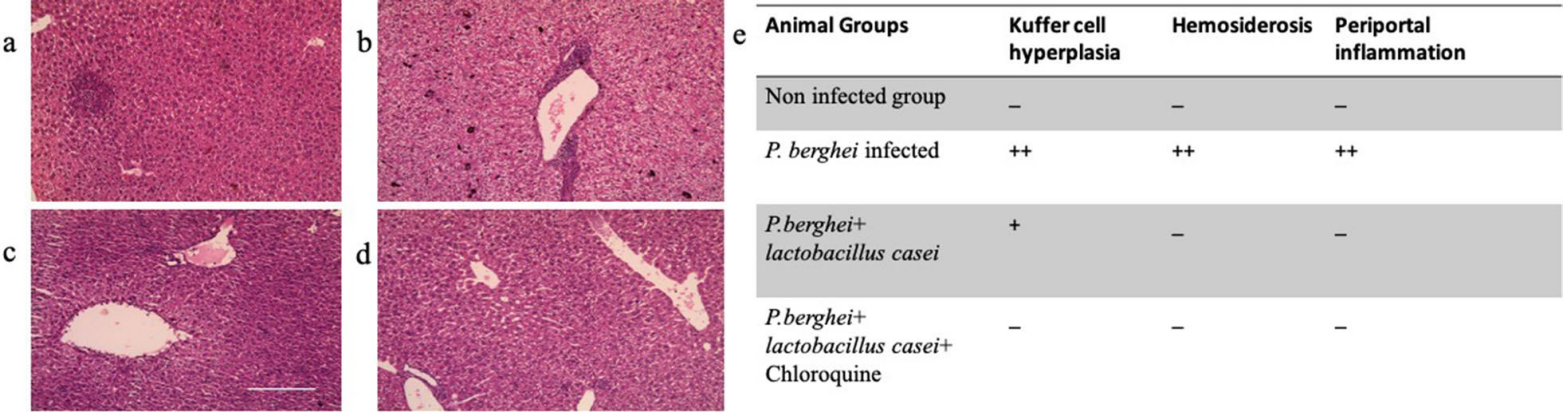
LS of control liver and treated liver under high magnification (200x). **a** Group I (Non-infected group): In this liver showing no periportal inflammation, hemosiderosis and Kuffer cell hyperplasia (**b**) Group II (*P. berghei* infected): Showing severe periportal inflammation with Kuffer cell hyperplasia and hemosiderosis (**c**) Group III (*P.berghei* + *L. casei*) liver: showing mild kuffer cell hyperplasia, periportal inflammation and traces of hemosiderosis (**d**) Group IV (*P. berghei* + *L. casei* + Chloroquine) liver: showing Kuffer cell hyperplasia, hemosiderosis and traces of periportal inflammation. **e** The scoring chart to show the effect of treatment on histopathological changes in the liver section

**Fig. 3:**
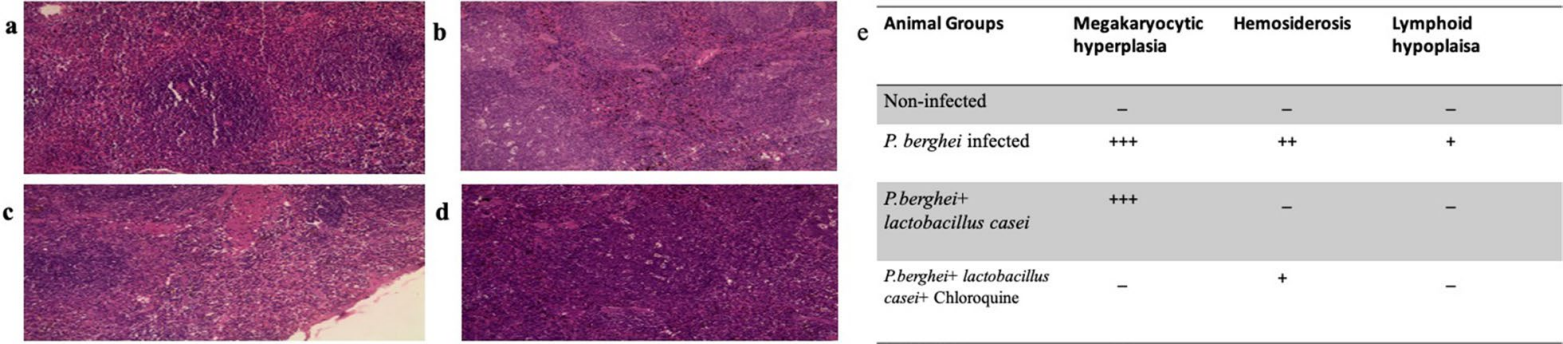
LS of Spleen under high magnification (200x). **a** Group l (Non-infected group) spleen (**b**) Group II (*P. berghei* infected) spleen: showing severe hemosiderosis, lymphoid hypoplasia and Megakaryocytic hyperplasia (**c**) Group III (*P. berghei* + *L. casei*) spleen: showing mild hemosiderosis and Megakaryocytic hyperplasia (**d**) Group IV (*P. berghei* + *L. casei* + Chloroquine) spleen: showing mild hemosiderosis, Megakaryocytic hyperplasia, and no lymphoid hypoplasia. **e** The scoring chart to show the effect of treatment on histopathological changes in the spleen section

There were no changes found in the case of a control group as it has shown normal pathology (Figs. 2a and 3a). Hemosiderosis and periportal inflammation in the liver section was found more in an infected group (2b) but there is a reduction of hemosiderosis and periportal inflammation when treatment of *L. casei* (2c) was given and further suppression was seen in the group treated with chloroquine and *L. casei* (2d). In the case of the spleen, megakaryocytic hyperplasia, lymphoid hypoplasia along with hemosiderosis have been seen in the infected group (3b). It has become mild and traces of hemozoin pigments were seen when *L. casei* treatment was given (3c) but these were further reduced in the chloroquine and *L. casei* treated group (3d).

### Discussion

This study was planned to show the synergistic effect of *L. casei* (Probiotic) along with chloroquine in malaria-induced mice. *L. casei* were given at 0.1 mL for 3 days. A similar study was conducted by Oyetayo et al*.* [10]*,* in which they showed *Lactobacillus acidophilus* and *L. casei* protective effects*.* The rat has been dosed with *Lactobacillus* and showed lowering of serum alanine aminotransferase activities which have values of 15.50 and 18.27 IU/L as compared to that of the control. Liver functions were improved by *L. casei* which was later confirmed by toxicological data of rat serum. In the present study, probiotic treatment along with conventional drug therapy showed statistically significant reduction in the parasitemia rate. There was an improvement in histopathological damages that are caused by the *plasmodium* parasite in organs like the liver and spleen. Hence, the present study confirmed that the use of probiotics as add-on therapy along with conventional drug therapy has beneficial effects. Chloroquine when given along with *L. casei* causes further decrease in the parasitemia count leading to maximum suppression of the parasite growth. Blood film microscopic examination was found to be lesser than 2% within the period of four days before starting treatment, while chloroquine + *L. casei* cleared parasitemia on the third day of treatment. A previous study done by Khalifa EA, 2016, has shown that as compared to the non-treated group the *L. casei* treated group has decreased the parasite load in the infected mice. So, probiotics can be considered as a promising and hopeful alternative for the treatment of various parasitic diseases. Different standard drug therapies in combination with probiotics can provide a definite treatment to eradicate the various parasitic infections [11]. Moreover, probiotics treatment has also been effective in treating bacterial infections as well, e.g., *Salmonella*. Probiotics have several mechanisms of action for example, it increases the production of acid which kills the acid-sensitive bacteria or it releases the bacteriocins that may inhibit the growth of other pathogenic bacteria [12]. Another study which has been conducted in mice infected with *Strongyloides venezuelensis,* shown a reduction in the number of worms (about 33%) and egg output upon giving probiotics and has also improved the immune responses. However, the factors responsible for these effects are still not clear [13]. On the other hand, the study which was done by Juliette Guitard et al., has shown that administration of *L.casei* mixture (daily) was unable to eradicate the complete parasite load (*Cryptosporidium parvum*) in the neonatal rat model. The reason for this may be due to the lack of production of INF-γ which could show the protective effect against the parasite [14].

This study has shown that when *L. casei* is given along with the standard drug therapy (chloroquine) it shows a synergistic effect in the mice model of malaria as it has reduced the parasitemia count and improved the pathological changes that appeared after getting the infection.

## Limitations

The study includes the only preliminary finding that shows the only effect of *L. casei* as one of the probiotics, on malaria parasite in *vivo* environment. However, it lacks to depict host response while taking probiotics in case of malaria infection. A more elaborated protocol is required for further deeper investigations such as studying of the involvement of innate and adaptive immunity through estimation of antibodies, T-subset regulation, and cytokines estimation. Additionally, experiments such as a survival plot would better explain the usefulness of these prophylactic measures.

## Supplementary Information

**Additional file 1: Figure S1. **Giemsa Stain slide of parasitemia on fourth day of treatment observed under 1000× magnification.

## Data Availability

All data generated or analyzed during this study are included in this published article and its supplementary information files.

## Abbreviations

ANOVA: Analysis of variance
ATCC: American type culture collection
IgA: Immunoglobulin A
IL-10: Interleukin-10
INF-γ: Interferon-gamma
IU/L: International units per liter
*L.casei*: *Lactobacillus casei*
PBS: Phosphate Buffer Saline
RBCs: Red blood corpuscles
SD: Standard deviation
SPSS: Statistical Package for the Social Sciences
