# Supplementary figures and images for: Evaluation of the effect of probiotic as add-on therapy with conventional therapy and alone in malaria induced mice
Source: BMC Res Notes. 2021 Jun 30;14:246. doi: 10.1186/s13104-021-05661-1 (PMC8244208; doi:10.1186/s13104-021-05661-1)

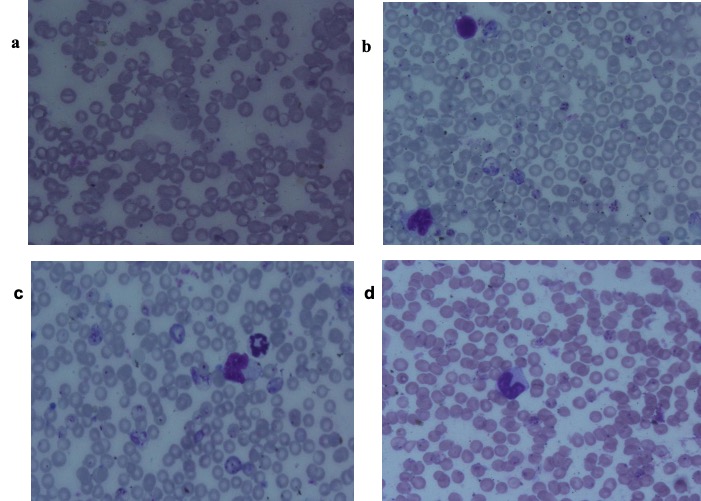

Supplement: Supplementary file 1 — Additional file 1: Figure S1. Giemsa Stain slide of parasitemia on fourth day of treatment observed under 1000× magnification. [file 13104_2021_5661_MOESM1_ESM.jpg]
